# Gastrointestinal Bleeding Associated With Nasojejunal Tube Injury in a Patient With Septic Shock: A Case Report

**DOI:** 10.7759/cureus.88691

**Published:** 2025-07-24

**Authors:** Zi-Cong Zheng, Xiao-Ling Wang, Li Fu

**Affiliations:** 1 Department of Clinical Nutrition, The Eighth Affiliated Hospital, Sun Yat-sen University, Shenzhen, CHN; 2 Department of Intensive Care Unit, The Eighth Affiliated Hospital, Sun Yat-sen University, Shenzhen, CHN

**Keywords:** enteral nutrition, gastrointestinal bleeding, nasojejunal tube, septic shock, type 2 diabetes mellitus

## Abstract

This report presents a rare case of GI bleeding caused by nasojejunal feeding in a patient with type 2 diabetes mellitus and septic shock, complicated by multiple organ dysfunction syndrome (MODS). The case analyzes the underlying causes of recurrent GI bleeding and discusses the prevention and management of mechanical complications associated with enteral nutrition (EN), particularly mucosal injury caused by nasojejunal tubes. It emphasizes the necessity of modifying EN protocols for high-risk patients and offers suggestions for improvement.

## Introduction

Nutritional status in critically ill patients, particularly those with septic shock, strongly influences clinical outcomes [1]. Despite a high risk of GI dysfunction due to immune/inflammatory responses, enteral nutrition (EN) remains the preferred nutritional intervention [2]. However, EN delivery via nasogastric or nasojejunal tubes carries a risk of mucosal membrane pressure injury (MMPI), with nasal mucosal involvement reported in up to 10.9% of ICU patients [3]. In contrast, data on GI mucosal injury, especially duodenal damage related to nasojejunal tubes, remain scarce, limiting understanding of such complications in septic shock [4].

This case report describes a patient with septic shock, multiple organ dysfunction syndrome (MODS), and type 2 diabetes mellitus who developed GI bleeding associated with nasojejunal tube-induced injury during nutritional support. The subsequent adjustment of the nutritional approach is presented, along with a review of relevant literature, aiming to highlight the importance of early recognition of this rare but clinically significant complication.

## Case presentation

Chief complaint​

A 58-year-old male with poorly controlled type 2 diabetes mellitus (20-year history) and a 40-year smoking history (20-40 cigarettes/day) presented with 4 days of fatigue and anorexia, followed by 12 hours of fever, dyspnea, and altered consciousness. He was transferred from an outside hospital and admitted to our ICU on March 1, 2022.

On admission​

The patient exhibited severe systemic inflammation and hemodynamic instability.

Vital Signs

Temperature 39.2°C, heart rate 150 bpm (tachycardia), respiratory rate 35 bpm (tachypnea), blood pressure 98/55 mmHg (hypotension); SpO₂ 99% on nasal oxygen.

General Appearance

Drowsy, poorly cooperative, with Kussmaul respirations (consistent with metabolic acidosis).

Physical Examination

Mild jaundice (skin/sclera); coarse lung sounds (no crackles); regular heart rhythm (no murmurs); soft, non-tender abdomen (no hepatorenal tenderness); no edema in lower limbs.

Key Laboratory Findings

Marked elevation of inflammatory markers (hs-CRP 175.34 mg/L, WBC count 16.74×10⁹/L, neutrophil percentage (NEU%) 94.4%, procalcitonin (PCT) >100.0 ng/mL); coagulopathy (prothrombin time (PT) 17.2 s, activated partial thromboplastin time (APTT) 48.6 s, D-dimer >20 μg/mL); multiple organ dysfunction (elevated bilirubin and liver enzymes, acute kidney injury with creatinine 358.6 μmol/L, cardiac troponin I 0.249 ng/mL); hypoalbuminemia (albumin 19.61 g/L) (Table 1).

**Table 1:** Laboratory test results on admission.

Test Name	Result	Reference Range	Units
Inflammatory Markers			
- High-sensitivity C-reactive protein (hs-CRP)	175.34	0-5	mg/L
- WBC count	16.74 × 10⁹	3.5-9.5 × 10⁹	/L
- Neutrophil percentage (NEU%)	94.4	40-75	%
- Hemoglobin (HGB)	90	130-175 (male) / 120-155 (female)	g/L
				- Platelet count (PLT)	32.00 × 10⁹	125-350 × 10⁹	/L
Liver and Renal Function			
- Total bilirubin (TBIL)	65.3	3.4-17.1	μmol/L
- Conjugated bilirubin (Bc)	39.6	0-6	μmol/L
- Albumin (ALB)	19.61	40-55	g/L
- Alanine aminotransferase (ALT)	248	7-40	U/L
- Aspartate aminotransferase (AST)	430	13-35	U/L
- Carbon dioxide combining power (CO₂-CP)	14	22-31	mmol/L
- Blood urea nitrogen (BUN)	23.62	2.8-7.1	mmol/L
- Creatinine (CR)	358.6	53-106 (male) / 44-97 (female)	μmol/L
				Cardiac Markers			
- Cardiac troponin I (cTnI)	0.249	≤0.04	ng/mL
- Myoglobin (MYO)	3293	25-72	ng/mL
- N-terminal pro-B-type natriuretic peptide (NT-proBNP)	4080	<125 (age <50), <450 (age 50-74) , <900 (age ≥75)	pg/mL
				Other Markers			
				- β-Hydroxybutyrate (β-HB)	0.57	0.03-0.30	mmol/L
- Procalcitonin (PCT)	>100.0	<0.05	ng/mL
- Interleukin-6 (IL-6)	56	<7	pg/mL
Coagulation Profile			
- Prothrombin time (PT)	17.2	11-14	s
- Activated partial thromboplastin time (APTT)	48.6	25-37	s
- D-Dimer (FEU)	>20	<0.5	μg/mL
- Fibrin degradation products (FDP)	>150	<5	μg/mL

Admission diagnoses

The patient was diagnosed with septic shock, multiple organ dysfunction syndrome (MODS), and type 2 diabetes mellitus.

Clinical management

Upon admission, the patient received rapid fluid resuscitation, volume expansion, blood transfusion, broad-spectrum antibiotics, and anticoagulation therapy. Organ support measures included vasopressors to maintain blood pressure, mechanical ventilation via endotracheal intubation, and continuous renal replacement therapy (CRRT) to stabilize the internal environment.

During treatment, the patient developed non-ST-elevation myocardial infarction (NSTEMI) and was managed with antiplatelet therapy. Liver abscess was identified as the source of infection, and percutaneous drainage was performed. His condition gradually stabilized following the above interventions.

A nasojejunal tube was placed on March 8 (Day 8 post-admission) to initiate enteral feeding. From March 14 (Day 14 post-admission) onward, the patient experienced recurrent episodes of gastrointestinal bleeding and underwent multiple endoscopic hemostatic procedures. Octreotide was also administered. On March 28 (Day 28 post-admission), bedside gastroscopy revealed mucosal injury and bleeding, likely caused by the nasojejunal tube. The feeding tube was subsequently removed.

During this period, intermittent endoscopic hemostasis was performed, and parenteral nutrition (PN) became the primary method of nutritional support. From April 20 (Day 51 post-admission) onward, no further gastrointestinal bleeding occurred, and the patient's vital signs remained stable. On April 25 (Day 56 post-admission), he was transferred to a general ward for continued treatment.

Nutritional therapy process

Nutritional Screening and Assessment

The total NRS-2002 score was 6, indicating high nutritional risk (Table 2).

**Table 2:** Nutritional risk screening (NRS-2002) assessment.

Assessment Component	Criteria	Score
1. Severity of Disease	APACHE II score = 21 (Corresponding to NRS-2002 criteria: severe disease, such as ICU admission or mechanical ventilation)	3
2. Nutritional Status	Comatose and unable to eat (Corresponding to NRS-2002 criteria: fasting ≥5 days or severe malnutrition)	3
3. Age	<70 years	0
Total Score	-	6

The total NRS-2002 score was 6, indicating high nutritional risk.

Early Nutritional Support

Early initiation of EN is a cornerstone of critical care, with evidence demonstrating improved outcomes in septic patients when initiated within 24-48 hours post-stabilization [5, 6]. In this case, the patient presented with septic shock (fever, hypotension, tachycardia) and was managed with fluid resuscitation and vasopressors. Following hemodynamic stabilization, a nasogastric tube was placed on March 4 (Day 4 post-admission), and EN was cautiously initiated at 10 mL/h of 5% glucose solution via slow pump infusion, concurrent with low-dose norepinephrine to maintain blood pressure, a known risk factor for gastrointestinal hypomotility. The formula was transitioned to a diabetes-specific polymer-based enteral formula after tolerance was confirmed.

Nutritional Therapy and Associated Complications

Despite the slow EN rate (10 mL/h), intermittent monitoring revealed persistent gastric residual volumes (GRVs) exceeding 200 mL. Prokinetic therapy with mosapride (10 mg tid via nasogastric tube) was initiated to enhance motility. However, by March 7, 24-hour GRV accumulated to 690 mL, surpassing the total EN volume administered (240 mL). Due to high nutritional risk (NRS-2002 score = 6) and prolonged hospitalization (>72 hours), refractory gastric retention (>500 mL/24 h despite prokinetics) prompted initiation of PN [6] on March 7 to ensure adequate caloric intake. Concurrently, postpyloric feeding was considered per guidelines [2]. The initial PN regimen is detailed in Table 3.

**Table 3:** Initial parenteral nutrition regimen (March 7, day 7 post-admission).

Component	Dosage	Units
Total Calories	1,036	kcal
Non-Protein Calorie-to-Nitrogen Ratio	102:1	-
Total Amino Acids	51	g
Administration Route	Central venous catheter	-

To facilitate EN, a nasojejunal tube was placed under endoscopic guidance on March 8 (Day 8 post-admission), and EN was resumed with the following regimen. As EN progressed, PN was gradually tapered and discontinued (Table 4).

**Table 4:** Enteral nutrition regimen (March 8, day 8 post-admission).

Component	Dosage	Units
Formula	Polymer-based enteral formula (diabetes-specific)	-
Total Calories	900	kcal
Volume	1,000	mL
Administration Route	Nasojejunal tube	-

On March 14 (Day 14 post-admission), the patient developed recurrent gastrointestinal bleeding, prompting suspension of EN, discontinuation of anticoagulants/antiplatelets, and initiation of acid suppression, blood transfusion, and fluid resuscitation.

**Figure 1:**
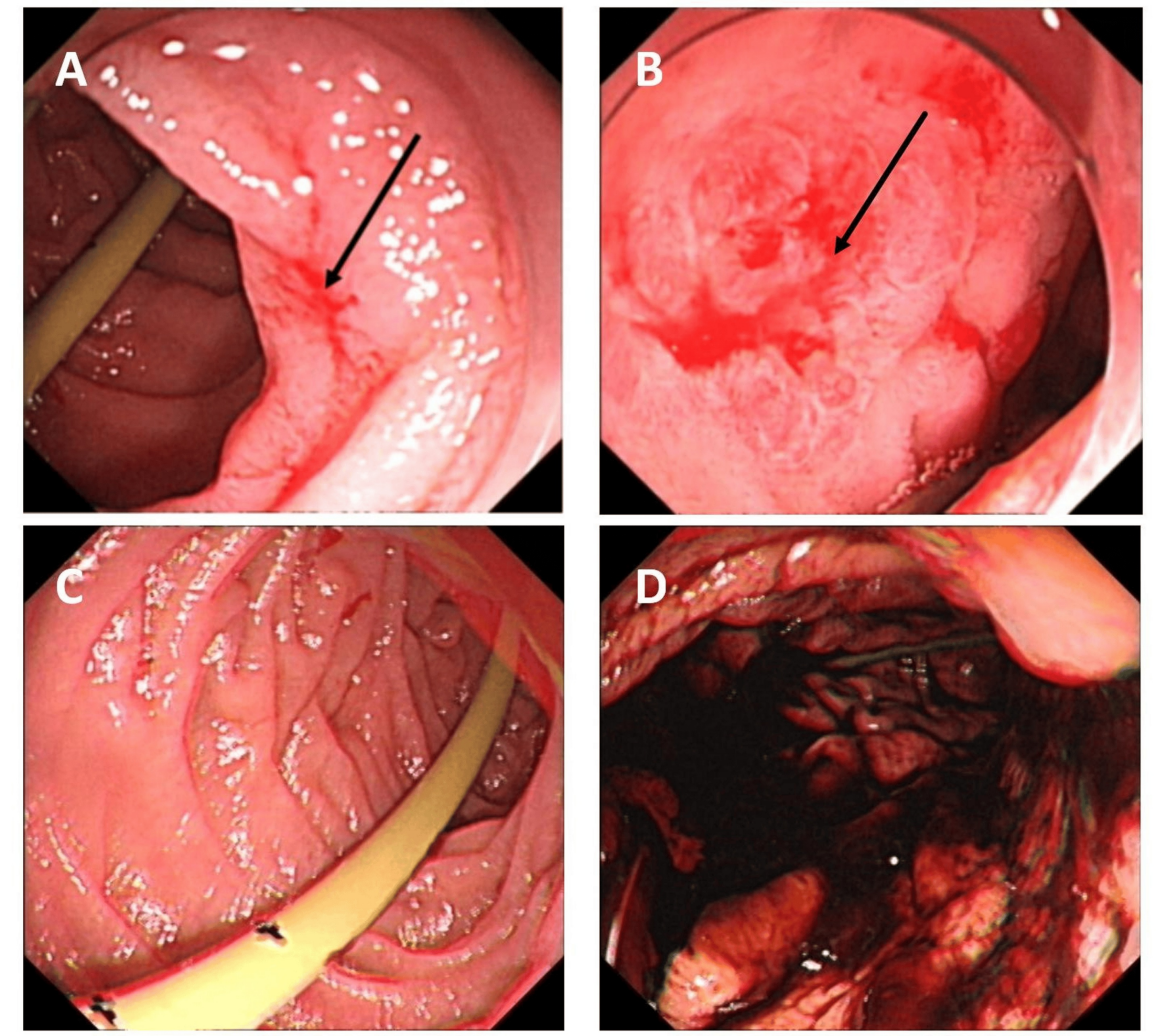
A-D: First gastroscopy on March 28, 2022. Multiple erosive lesions with fresh oozing were observed in the duodenal bulb and descending portion.

Fourteen days later, on March 28 (Day 28 post-admission), massive tarry stools and hypotension (75/57 mmHg) signaled acute GI bleeding with hypovolemic shock, confirmed by hemoglobin 68 g/L. Bedside gastroscopy revealed multiple duodenal erosions with active bleeding (Figure 1), managed with submucosal injections and octreotide. Due to failed EN tolerance and ongoing high nutritional risk, PN was reinstated with a revised regimen (Table 5: 1,295 kcal, 63.75 g protein).

**Table 5:** Revised parenteral nutrition regimen (March 28, day 28 post-admission).

Component	Dosage	Units
Total Calories	1,295	kcal
Non-Protein Calorie-to-Nitrogen Ratio	102:1	-
Total Amino Acids	63.75	g
Administration Route	Central venous catheter	-

On March 31 (Day 31 post-admission), intermittent melena recurred. Repeat gastroscopy showed bleeding ulcers and a longitudinal ulcer suspicious for tube compression injury. Hemostasis was achieved via titanium clips and epinephrine injections, and the nasojejunal tube was removed.

**Figure 2:**
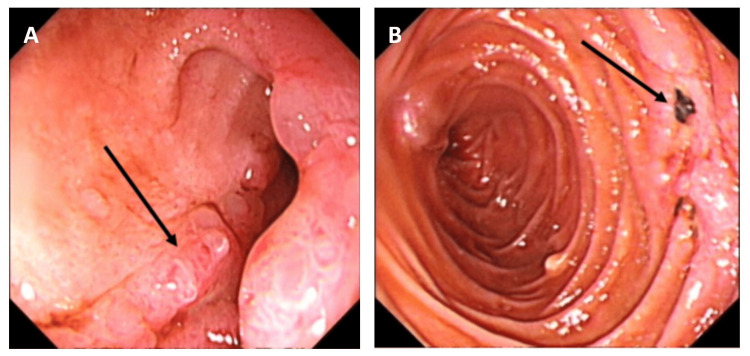
A-B: Third gastroscopy on April 8, 2022. A: A longitudinal ulcer scar was observed in the duodenal bulb. B: An ulcer in the descending portion was covered with a black blood clot and showed mild peripheral oozing.

On April 8 (Day 39 post-admission), a third gastroscopy showed multiple erosions in the descending duodenum without active bleeding. A longitudinal ulcer in the bulb was healing, while one descending ulcer had black clots with mild oozing (Figure 2), managed by diluted epinephrine injection.

**Figure 3:**
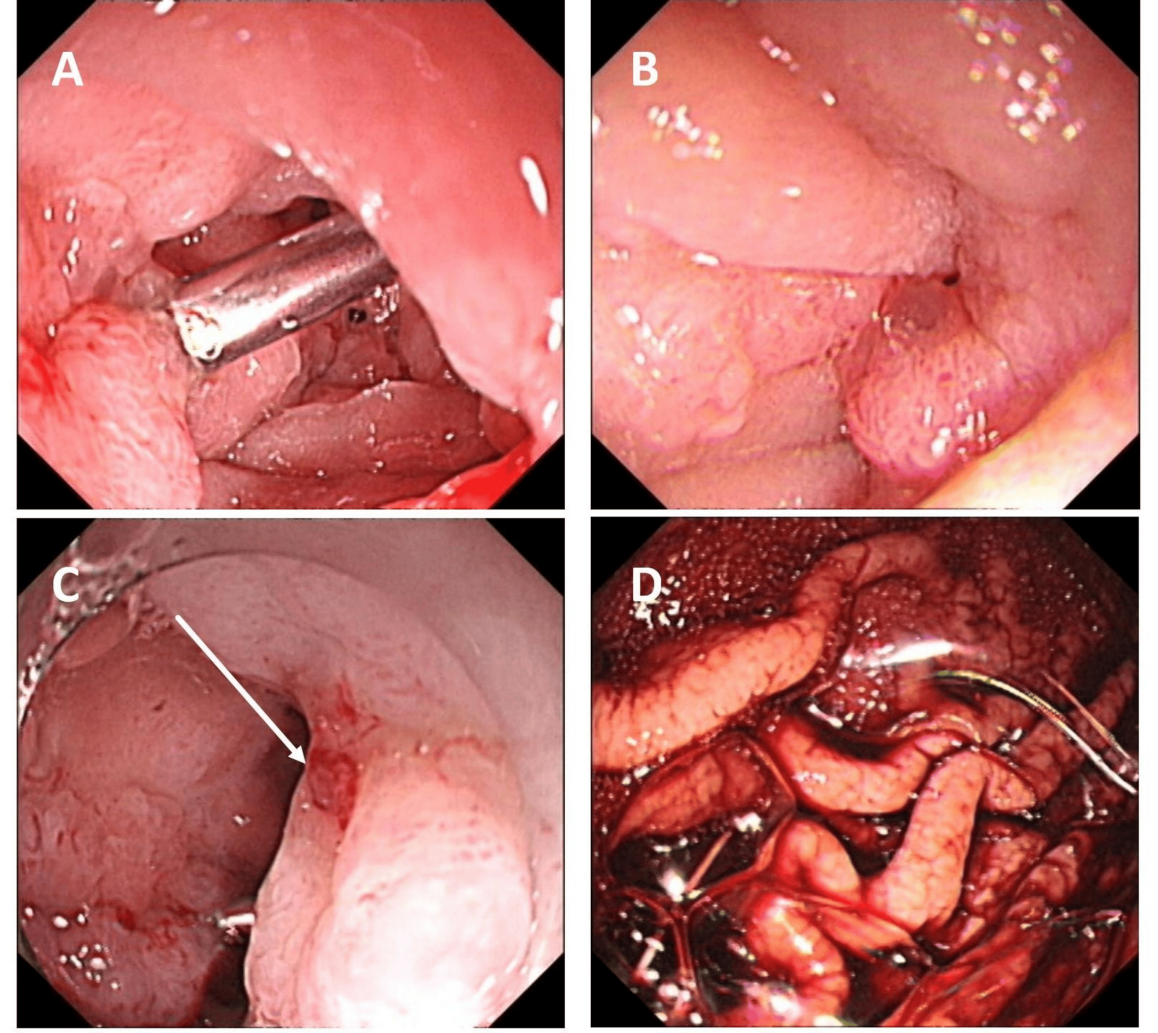
A-D: Fourth gastroscopy on April 13, 2022. An ulcer with a visible vascular stump was noted on the posterior wall of the duodenal bulb. Multiple erosions were present at the bulb-descending junction, and another ulcer was observed in the descending portion.

On April 13 (Day 44 post-admission), a fourth gastroscopy revealed a posterior wall bulb ulcer with a visible vascular stump (no active bleeding), treated with local epinephrine. Erosions at the bulb-descending junction and a descending ulcer were noted, with normal colonoscopy findings (Figure 3). Concurrently, PN was adjusted to the regimen in Table 6, reflecting a 16.1% increase in caloric support and a 29.4% higher non-protein calorie-to-nitrogen ratio to optimize recovery from ulcer healing.

**Table 6:** Parenteral nutrition regimen (April 13, day 44 post-admission).

Component	Dosage	Units
Total Calories	1,504	kcal
Non-Protein Calorie-to-Nitrogen Ratio	132:1	-
Total Amino Acids	63.75	g
Administration Route	Central venous catheter	-

**Figure 4:**
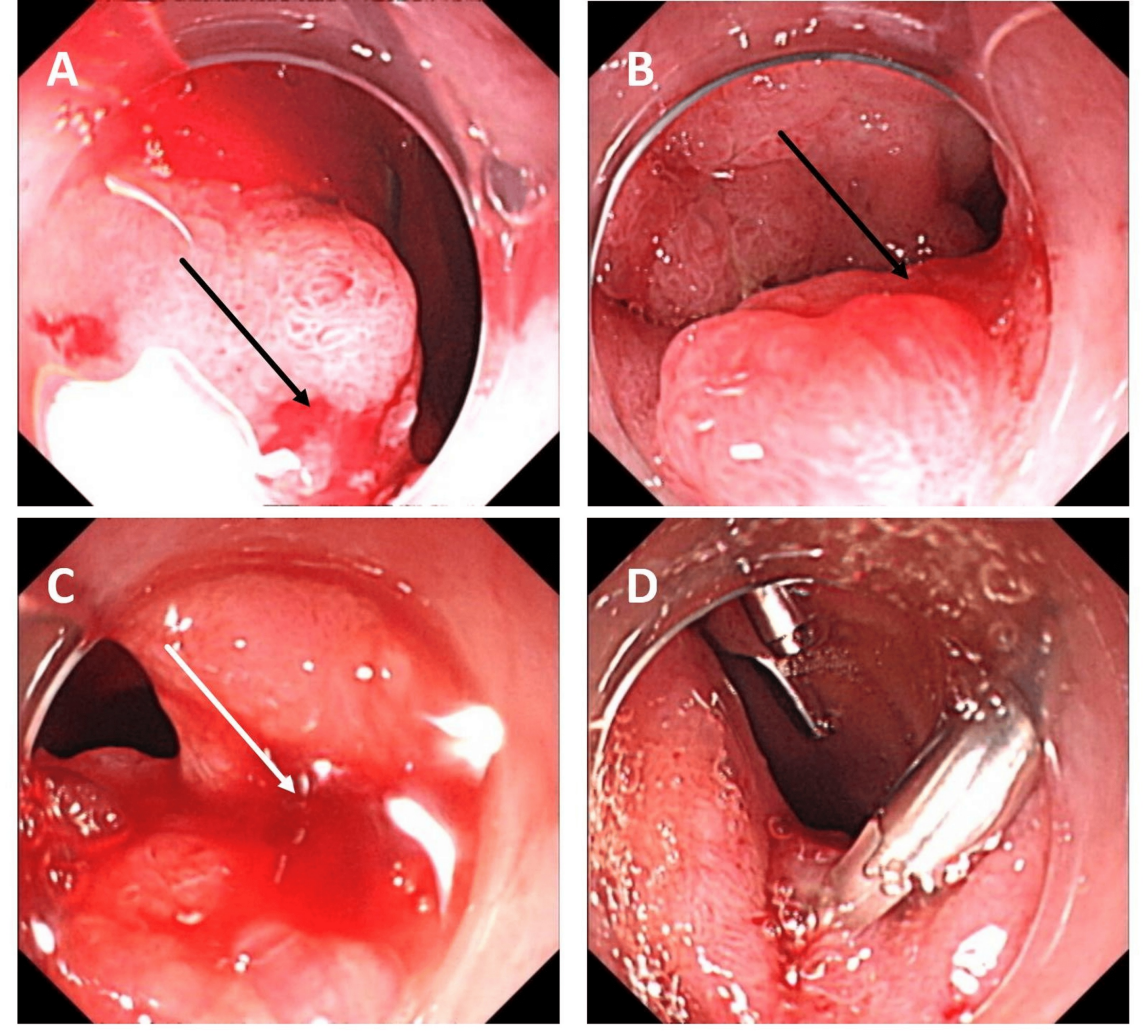
A-D: Fifth gastroscopy on April 17, 2022. Multiple erosive lesions with active bleeding were observed at the bulb-descending junction of the duodenum.

Four days later, on April 17 (Day 48 post-admission), a fifth gastroscopy identified active bleeding from multiple descending duodenal erosions (Figure 4), controlled by norepinephrine injection and titanium clips. By April 20 (Day 51 post-admission), bleeding resolved, gastric retention improved, and oral 5% glucose was initiated. On April 25 (Day 56 post-admission), stable vital signs allowed transfer to the general ward with a nasogastric tube and liver abscess drain in place.

Nutritional support was tailored dynamically to the clinical status during the treatment period. EN commenced on March 4 at 240 kcal/day. Due to intolerance, it was discontinued and replaced with PN on March 7 (1,036 kcal/day). EN was briefly resumed on March 8 at 900 kcal/day. On March 28, amid hemorrhagic shock, PN was increased to 1,295 kcal/day to counteract acute catabolism, though total protein (46.2 g/L) and albumin (25.26 g/L) remained low due to ongoing bleeding. Following nasojejunal tube removal on March 31, PN was further escalated to 1,504 kcal/day on April 13. This coincided with a gradual stabilization of nutritional indicators, with total protein rising from 54.8 to 60.8 g/L. This progression confirms that intensifying nutritional support, coupled with bleeding control achieved post-tube removal, was essential for mitigating the early deterioration (Table 7).

**Table 7:** Trends in hematology, coagulation, nutrition, and intake following initial GI bleed. Hb: Hemoglobin; HCT: Hematocrit; PLT: Platelet Count; PT: Prothrombin Time; INR: International Normalized Ratio; TP: Total Protein; Alb: Albumin; EN: Enteral Nutrition; PN: Parenteral Nutrition.

Time Point	Key Laboratory Findings	Nutrient Intake
March 14 (First GI bleeding)	Hb 65 g/L, HCT 17.6%, PLT 85×10⁹/L; PT 13.6 s, INR 1.06	EN, 900 kcal
March 28 (Hemorrhagic shock)	Hb 62 g/L, HCT 19.4%, PLT 55×10⁹/L; TP 46.2 g/L, Alb 25.26 g/L	PN, 1,295 kcal
March 29 (1 day after hemostasis)	PLT 87×10⁹/L; PT 17.6 s, INR 1.49	PN, 1,295 kcal
March 31 (Nasojejunal tube removal)	Hb 70 g/L, HCT 20.8%, PLT 81×10⁹/L; TP 54.8 g/L, Alb 29.64 g/L; PT 15.8 s, INR 1.29	PN, 1,295 kcal
April 8 (Endoscopic healing confirmation)	Hb 84 g/L, HCT 24.6%, PLT 161×10⁹/L; PT 14.5 s, INR 1.15	PN, 1,295 kcal
April 14 (1 day after parenteral nutrition escalation)	TP 51.69 g/L, Alb 30.72 g/L	PN, 1,504 kcal
April 20 (Bleeding cessation)	Hb 91 g/L, HCT 27.2%, PLT 95×10⁹/L	PN, 1,504 kcal
April 25 (Transfer to general ward)	TP 60.8 g/L, Alb 28.8 g/L	PN, 1,504 kcal

## Discussion

Common causes of GI bleeding and characteristics of this case

Upper GI bleeding is often precipitated by factors such as a history of prior GI bleeding, anticoagulant use, high-dose nonsteroidal anti-inflammatory drugs (NSAIDs), and advanced age [7]. Systemic comorbidities, including stroke and shock, also substantially elevate the risk [8]. In this case, the patient presented with multiple high-risk factors for GI bleeding: septic shock, MODS, treatment for non-ST-segment elevation myocardial infarction (NSTEMI) involving anticoagulants and antiplatelet agents, and long-standing, poorly controlled type 2 diabetes mellitus. The bleeding risk was further amplified by chronic diabetes-induced vascular damage, exacerbated by malnutrition, as well as platelet dysfunction and coagulopathy secondary to renal insufficiency. Additionally, diabetes-associated microvascular injury, particularly in the context of poor glycemic control, likely contributed significantly to the overall bleeding susceptibility [8-11].

Notably, stress-related mucosal disease (SRMD), commonly referred to as stress ulcers, is a well-recognized cause of upper GI bleeding in critically ill patients. These lesions typically localize to the gastric fundus and body, and present as diffuse subepithelial hemorrhages or erosions, and are most prevalent within 72 hours of critical illness onset [11]. In contrast, the present case featured delayed bleeding on post-admission day 14, well beyond the typical SRMD window, and was endoscopically identified as a longitudinal duodenal ulcer corresponding to the nasojejunal tube trajectory. This focal lesion morphology and location are inconsistent with SRMD's characteristic diffuse gastric pathology.

Despite comprehensive management, including endoscopic intervention, nutritional optimization, and pharmacotherapy adjustment, recurrent hemorrhage persisted, highlighting the synergistic effects of the underlying systemic pathophysiology: septic shock and MODS induced a coagulopathy resembling acute traumatic coagulopathy (TIC), characterized by hyperfibrinolysis and clotting factor consumption [12, 13]; acute liver failure impaired synthesis of vitamin K-dependent factors (II, VII, IX, X); renal failure caused uremic platelet dysfunction, evidenced by thrombocytopenia (nadir 32 × 10⁹/L on admission, recovering to 161 × 10⁹/L by April 8) and impaired coagulation (PT 17.6 s, INR 1.49 on March 29) [13]; poorly controlled diabetes exacerbated hyperglycemia, which impeded ulcer healing through impaired collagen synthesis and sustained inflammation [14].

Prevention and management of mechanical complications due to EN

Enteral feeding tube placement can cause mechanical mucosal injury, potentially leading to ulceration and bleeding. In our case, the March 28, 2022, gastroscopy revealed multiple duodenal ulcers, including a longitudinal ulcer in the bulb with a pressure mark consistent with the tube shape. The subsequent healing of this ulcer after tube removal (documented by the April 8 gastroscopy) strongly supports the association between tube-induced mechanical pressure and recurrent mucosal injury.

When comparing our case with previous reports of mucosal ulceration related to feeding tubes, notable differences emerge. Most previous studies focused on patients with nasogastric tubes, while our case involved a nasojejunal tube. Additionally, the duration of tube placement in our case was relatively long, which may have contributed to more severe mechanical damage. In contrast to studies that primarily reported on patients without complex comorbidities, our patient had multiple underlying conditions, such as diabetes and liver failure, which likely increased mucosal fragility and impaired the healing process [15, 16]. These differences highlight the need for careful consideration of patient-specific factors when using enteral feeding tubes.

Epidemiologically, while most research on feeding tube complications focuses on blockage or dislodgement [15], comprehensive data on nasojejunal tube-related GI mucosal injury is scarce. A study showed submucosal hemorrhages and erosions at the esophagogastric junction within 24 hours of nasogastric tube insertion, indicating inherent mucosal injury risks [16]. Although nasojejunal tube-related complications are rare, in this patient, the combination of tube-induced mechanical trauma, mucosal fragility, and impaired healing likely contributed to persistent bleeding.

Improvements in EN strategies

Common EN complications like gastric retention and diarrhea can be mitigated through proper patient positioning, infusion control, formula temperature management, and energy optimization [17, 18]. Evidence supports early EN initiation (within 27 hours) without increasing rebleeding risk in GI bleeding patients [19]. However, our patient experienced recurrent bleeding during EN, which was ultimately linked to nasojejunal tube-induced duodenal ulcers.

For future nutritional management of high-risk patients with similar conditions, an individualized strategy could be considered. For instance, if reintroducing EN, a softer and more flexible nasojejunal tube with a smaller diameter could be selected to reduce mechanical pressure on the mucosa. The infusion rate should be gradually increased, starting from a low flow rate of 10-20 mL/h, and closely monitored for any signs of mucosal injury or bleeding. Regular endoscopic examinations should be scheduled to assess the condition of the GI mucosa.

The transition from EN to PN after tube removal significantly reduced bleeding episodes, underscoring the importance of individualized nutritional strategies in high-risk patients.

## Conclusions

This case highlights the need for heightened vigilance during EN in high-risk patients with upper GI bleeding. Key observations include the association between nasojejunal tube-induced pressure injury and recurrent duodenal bleeding, underscoring the importance of prompt endoscopic evaluation at the first sign of bleeding, with particular focus on the often-overlooked posterior wall of the duodenal bulb and post-bulbar regions for feeding tube-related mucosal lesions.

Clinically, this mandates integrated strategies: (1) immediate endoscopic identification of bleeding sites with targeted hemostasis; (2) adaptive medication adjustments (e.g., anticoagulants, antiplatelets) based on evolving coagulation status; and (3) consideration of narrower, softer feeding tubes to mitigate mechanical injury in patients with coagulation abnormalities. These measures aim to balance nutritional support with minimized risk of tube-related complications in vulnerable populations.

## References

[1] Park S, Park SH, Kim Y, Lee GH, Kim HS, Lim SY, Choi SA (2023). Optimal nutritional support strategy based on the association between modified NUTRIC score and 28-day mortality in critically ill patients: a prospective study. Nutrients.

[2] Singer P, Blaser AR, Berger MM (2023). ESPEN practical and partially revised guideline: clinical nutrition in the intensive care unit. Clin Nutr.

[3] Chen G, Li X, Li X, Liu S, Xie J (2024). Mucosal membrane pressure injury in intensive care units: a scoping review. Intensive Crit Care Nurs.

[4] Fulbrook P, Lovegrove J, Butterworth J (2023). Incidence and characteristics of hospital-acquired mucous membrane pressure injury: a five-year analysis. J Clin Nurs.

[5] Moon SJ, Ko RE, Park CM, Suh GY, Hwang J, Chung CR (2023). The effectiveness of early enteral nutrition on clinical outcomes in critically ill sepsis patients: a systematic review. Nutrients.

[6] Nutrition CSOP (2025). Guidelines for medical nutritional therapy in adult sepsis patients. Zhonghua Yi Xue Za Zhi.

[7] Wilkins TM, Wheeler BB, Carpenter MB (2020). Upper gastrointestinal bleeding in adults: evaluation and management. Am Fam Physician.

[8] Crooks CJ, West J, Card TR (2013). Comorbidities affect risk of nonvariceal upper gastrointestinal bleeding. Gastroenterology.

[9] Nathan DM, Lachin JM, Bebu I (2022). Glycemia reduction in type 2 diabetes - microvascular and cardiovascular outcomes. N Engl J Med.

[10] Horton WB, Barrett EJ (2021). Microvascular Dysfunction in Diabetes Mellitus and Cardiometabolic Disease. Endocr Rev.

[11] Bardou M, Quenot JP, Barkun A (2015). Stress-related mucosal disease in the critically ill patient. Nat Rev Gastroenterol Hepatol.

[12] Maier CL, Brohi K, Curry N (2024). Contemporary management of major haemorrhage in critical care. Intensive Care Med.

[13] Simmons JW, Powell MF (2016). Acute traumatic coagulopathy: pathophysiology and resuscitation. Br J Anaesth.

[14] Ko KI, Sculean A, Graves DT (2021). Diabetic wound healing in soft and hard oral tissues. Transl Res.

[15] Metheny NA, Meert KL, Clouse RE (2007). Complications related to feeding tube placement. Curr Opin Gastroenterol.

[16] Motta AP, Rigobello MC, Silveira RC, Gimenes FR (2021). Nasogastric/nasoenteric tube-related adverse events: an integrative review. Rev Lat Am Enfermagem.

[17] Hoffmann M, Schwarz CM, Fürst S, Starchl C, Lobmeyr E, Sendlhofer G, Jeitziner MM (2020). Risks in management of enteral nutrition in intensive care units: a literature review and narrative synthesis. Nutrients.

[18] Wanden-Berghe C, Patino-Alonso MC, Galindo-Villardón P, Sanz-Valero J (2019). Complications associated with enteral nutrition: CAFANE study. Nutrients.

[19] Zhang H, Wang Y, Sun S (2019). Early enteral nutrition versus delayed enteral nutrition in patients with gastrointestinal bleeding: a PRISMA-compliant meta-analysis. Medicine (Baltimore).

